# 

**Published:** 1876-10

**Authors:** 


[Vol. XVI] OCTOBER, 1876. [No. 3.]

### BUFFALO

### Medical and Surgical Journal.

S.G.O.L.
INDEXED

EDITED BY EDWARD N. BRUSH, M. D.

BUFFALO:

PUBLISHED FOR THE EDITOR.

1876.

